# Perioperative blood pressure management with clevidipine during coiling of cerebral artery aneurysms

**DOI:** 10.4103/1658-354X.57880

**Published:** 2009

**Authors:** Thomas Meyer, Joseph D. Tobias

**Affiliations:** 1 *Department of Anesthesiology, University of Missouri, Missouri, Colombia*; 2 *Department of Pediatrics, University of Missouri, Missouri, Colombia*

**Keywords:** *Clevidipine*, *cerebral aneurysm*, *cerebral coiling*

## Abstract

The tight control of blood pressure (BP) is mandatory during cerebral aneurysm coiling to minimize abrupt changes in the transmural pressure across the aneurysm and thereby decrease the risk of rupture. Critical times during these procedures when significant BP changes may occur include anesthetic induction, endotracheal intubation, and emergence. Clevidipine is a recently introduced, rapidly acting dihydropyridine calcium channel antagonist. Its rapid metabolism by tissue and plasma esterases results in an effective half-life of 1 to 3 minutes. We present our preliminary experience with the use of clevidipine to control BP during the anesthetic care of three patients undergoing coiling of cerebral aneurysms in the interventional radiology suite.

## INTRODUCTION

The tight control of mean arterial pressure (MAP) is mandatory during cerebral aneurysm coiling to minimize abrupt changes in the transmural pressure across the aneurysm and thereby decrease the risk of rupture. Critical times during these procedures when significant fluctuations in blood pressure (BP) may occur include anesthetic induction, endotracheal intubation, and emergence. Several pharmacologic agents have been employed to avoid these abrupt changes in MAP including sodium nitroprusside, nitroglycerin, nicardipine, hydralazine, and labetalol. Clevidipine is a recently introduced, rapidly acting dihydropyridine calcium channel antagonist. Its rapid metabolism by tissue and plasma esterases results in an effective half-life of 1 to 3 minutes. We present our preliminary experience with the use of clevidipine to control MAP during the anesthetic care of two patients undergoing coiling of cerebral aneurysms in the interventional radiology suite.

## CASE REPORTS

Review of these patients' medical records and presentation of these cases in this format was approved by the Institutional Review Board of the University of Missouri.

### Case 1

The first patient was a 41-year-old, 68 kilogram man with a past medical history of asthma, smoking, and hepatitis. The patient was found to have a right internal carotid artery terminus aneurysm by magnetic resonance imaging which was performed during an evaluation for recurrent headaches. The patient had no previous history of cerebral vascular accident (CVA) or subarachnoid hemorrhage (SAH). On the day of surgery, his preoperative vital signs included a BP of 111/64 mmHg and a heart rate (HR) of 59 beats/minute. The treatment plan was for coiling of the cerebral aneurysm in the interventional radiology suite under general anesthesia. Premedication included midazolam (2 mg) and fentanyl (50 μg) followed by placement of an arterial cannula in the right radial artery. The patient was transported to interventional radiology and positioned for comfort. Standard American Society of Anesthesiologists (ASA) monitors were placed. The initial BP was 100/62 mmHg with a HR of 94 beats/ minute. Following pre-oxygenation with 100% oxygen, anesthesia was induced with lidocaine (100 mg), fentanyl (200 μg), and propofol (total dose 150 mg). Endotracheal intubation was facilitated with vecuronium (10 mg). After endotracheal intubation, the BP was 105/52 mmHg with a HR of 71 beats/minute. The vital signs remained within 20% of baseline throughout the procedure with general anesthesia being maintained with desflurane (expired concentration 4-8%) and 250 μg of fentanyl. The procedure lasted approximately one hour and forty-five minutes. The aneurysm was only partially occluded thereby mandating the need to avoid hypertension during anesthetic emergence. Residual neuromuscular blockade was reversed with neostigmine and glycopyrrolate, lidocaine (100 mg) was administered, and the desflurane was discontinued. The systolic blood pressure (sBP) increased to 140-150 mmHg. A clevidipine infusion was started at 8 mg/hr with a decrease in the sBP to 120 mmHg within 2-3 minutes. The clevidipine infusion was decreased to 4 mg/hr and the sBP remained at 120 mmHg along. HR increased from 72 to 80 beats/minute following the start of the clevidipine during emergence. The patient's trachea was extubated and he was transported to the post-anesthesia care unit (PACU) with the clevidipine infusion at 4 mg/hr. His sBP remained at 110-120 mmHg. The clevidipine infusion was slowly weaned over the next 20 in the PACU in 2 mg/hr increments every 10 minutes. The patient was discharged home the next day without complications.

### Case 2

The second patient was an 83-year-old, 76 kg man with a past medical history of hypertension treated with lisinopril and hydrochlorothiazide and a distant history of tobacco use. During an evaluation for headaches, an MRI revealed an aneurysm of the left internal carotid artery (LICA) in the supraclinoid territory. There was no history of previous CVA or SAH. The morning of surgery, the patient's BP was 134/51 mmHg and the HR was 71 beats/minute. The patient had not taken his routine antihypertensive medications the morning of surgery. The treatment plan was for coiling of LICA aneurysm in the interventional radiology suite under general anesthesia. Preoperatively, an 18 gauge IV cannula and premedication provided by intravenous midazolam (2 mg). An arterial cannula was placed in the right radial arterial. The patient was transported to the interventional radiology suite and positioned to comfort. Standard ASA monitors were applied and the patient was pre-oxygenated with 100% oxygen. Anesthesia was induced with lidocaine (100 mg), fentanyl (100 μg), and propofol (total dose of 80 mg). Neuromuscular blockade was provided by vecuronium (10 mg). Immediately after endotracheal intubation, the BP was 105/70 mmHg with a HR of 60 beats/minute. Anesthesia was maintained with desflurane (expired concentration 4-6%) and 100 mg of fentanyl. The procedure lasted approximately one hour and twenty minutes. The vital signs remained within 20% of baseline throughout the procedure. Secondary to the anatomy of the aneurysm, coiling could not be accomplished. The patient's trachea was extubated during spontaneous ventilation with an expired concentration of 1 MAC of desflurane. Following tracheal extubation, the desflurane was discontinued. The SBP increased to 150-170 mmHg with a heart rate of 80-90 beats/minute. A clevidipine infusion was started at 2 mg/hour. Within 5-7 minutes, the sBP decreased to the 110-120 mmHg with an increase in the HR to 90-94 beats/minute. The patient was transferred to PACU with the clevidipine infusion at 2 mg/hr. The infusion was weaned over the next 30 minutes in 1 mg/hr increments in the PACU. He remained stable with the sBP ranging from 110-120 mmHg. The patient was discharged home the next day without complications.

### Case 3

The third patient was a 72-year-old, 85 kg man that found to have an ACOM aneurysm during a preoperative evaluation for an elective orthopedic operation. A CT scan of the head was obtained during the preoperative evaluation as the patient had neurologic symptoms similar to a transient ischemic attack. The patient's past medical history included a CVA in 2003 without residual neurologic deficit, osteoarthritis of the left shoulder and hypertension which was being treated with amlodipine and lisinopril. The treatment plan was to coil the aneurysm in the interventional radiology suite. On the morning of surgery, the patient did not take his usual anti-hypertensive medications. The preoperative vital signs on the morning of the procedure included a BP of blood 147/97 mmHg and a HR of 69 beats/minute. A 16 gauge intravenous cannula was started and following sedation with midazolam (2 mg) and fentanyl (100 μg), an arterial cannula was placed in the right radial artery. The patient was transported to the interventional radiology suite where standard ASA monitors were applied. Following preoxygenation, anesthesia was induced with lidocaine (100 mg), fentanyl (150 μg), and a total of 130 mg of propofol titrated to the BP. Neuromuscular blockaded was achieved with cisatricurium (20 mg). Following endotracheal intubation, anesthesia was maintained with maintained with isoflurane (end-tidal concentration of 0.5-1%) and fentanyl (total dose of 500 μg). Throughout the initial 2 hours of the procedure, the sBP varied from 100-120 mmHg with a diastolic BP of 70-85 mmHg. A clevidipine infusion was started at 2 mg/hour to control an increasing sBP up to 130-150 mmHg. The sBP returned to the range of 100-120 mmHg within 5 minutes of starting the infusion. The clevidipine infusion was continued for the next 90 minutes and the BP remained at 100-120 mmHg. A spike in the BP occurred with an increase of the sBP to 144 mmHg as the radiologist noted that there was bleeding from an intracranial vessel leading to the aneurysm (not the aneurysm itself) which occurred during the attempted coiling. Protamine was given to reverse heparin and the clevidipine infusion was increased 3 mg/hour. The sBP returned to its baseline value of 100-120 mmHg within 5 minutes. A CT scan of the head was obtained which demonstrated a subarachnoid hemorrhage with no change in the aneurysm. The patient was transported to the ICU with the clevidipine infusion at 3 mg/hr. This was continued for 4-6 hours in the ICU and then the patient was transitioned back to his routine oral anti-hypertensive medications and to oral nimodipine for preventing of cerebral vasospasm. His trachea was extubated the next day. The remainder of his post-procedure course was unremarkable without evidence of cerebral vasospasm or new neurologic deficit.

## DISCUSSION

In our 3 patients with cerebral artery aneurysms, we found that clevidipine effectively controlled BP intraoperatively and/or during emergence from general anesthesia with only a modest reflex increase in HR of 8-10 beats/ minute thereby preventing alterations in the transmural pressure of the aneurysm. Effective BP control was mandated since occlusion of the aneurysms could not be accomplished during the interventional procedure as well as the new SAH in our final patient. Although BP control may be problematic during both anesthetic induction and emergence, the various anesthetic agents used during the induction and maintenance phases can be titrated to limit BP changes.[1,2] However, these agents are discontinued during emergence thereby making this phase a more problematic period in regards to BP control. During emergence, we found that clevidipine could be rapidly titrated and used to effectively control BP. Although we chose to perform a deep extubation in our second patient, hypertension still occurred after the trachea was extubated when the desflurane was discontinued. Given the current recommendations to hold specific agents on the morning of surgery (angiotensin converting enzyme inhibitors or angiotensin receptor blocking agents) given their potential to result in refractory intraoperative hypotension,[3,4] intravenous agents may be necessary for perioperative BP control in such patients.

Options for perioperative BP control include sodium nitroprusside (SNP), labetolol, and nicardipine. SNP is a direct-acting, non-selective peripheral vasodilator that dilates resistance and capacitance vessels. Its rapid onset of action (approximately 30 seconds) allows its titration by continuous intravenous infusion for the control of perioperative BP. With the discontinuation of the infusion, BP rapidly returns to baseline values within minutes. Despite its uniform efficacy in BP control, its adverse effect profile may be problematic including the potential for excessive hypotension even when used within recommended dosing guidelines or even cardiovascular collapse with inadvertent overdosing, reflex tachycardia with risks for myocardial ischemia in patients with co-morbid cardiac disease, activation of the sympathetic nervous system, and rebound hypertension when the infusion is discontinued. With prolonged use, additional issues include tachyphylaxis and the potential for cyanide and thiocyanate toxicity. The potential for hypotension mandates intra-arterial BP monitoring. Labetolol is a competitive antagonist of the α_1_, β_1_, and β_2_-adrenergic receptors. With intravenous administration, initial BP effects can be seen within 2-5 minutes with a peak effect at 5-15 minutes. However, it has a relatively prolonged duration of action of 2-4 hours. Adverse effects may also be seen related to blockade of the β-adrenergic receptors including bradycardia, heart block, and bronchospasm. Furthermore, it is contraindicated in patients with depressed left ventricular function. Nicardipine is a calcium channel antagonist of the dihydropyridine group that vasodilates the systemic, cerebral and coronary vasculature with limited effects on myocardial contractility and stroke volume. Several case series have demonstrated that nicardipine is an effective agent for BP control in many clinical scenarios including the perioperative period.[5] Although its adverse effect profile including the potential for hypotension is limited, its duration of action may be prolonged following a continuous infusion.

Clevidipine is an ultra-shorting acting, intravenous calcium channel antagonist of the dihydropyridine class which is metabolized by non-specific blood and tissue esterases.[6] To date, the majority of clinical experience with its use has been in the adult cardiac surgery population. In a prospective trial of 152 adult cardiac surgery patients, clevidipine effectively controlled BP (defined as a reduction of sBP by ≥15%) in 92.5% of patients compared to 17.3% of placebo patients (failure rate of 82.7% in placebo-treated patients).[7] Target BP control was achieved in a median time of 6 minutes (95% confidence interval of 6-8 minutes). HR increased from a baseline of 71 beats/minute to a maximum value of 84 beats/minute. There were no differences between clevidipine and placebo in regards to the adverse effect profile. When comparing clevidipine with SNP, nitroglycerin or nicardipine for the treatment of acute hypertension in adult cardiac surgery patients, Aronson *et al*., reported no difference in the incidence of stroke, myocardial infarction or renal dysfunction among the treatment groups.[8] However, BP control was more effective with clevidipine when compared with nitroglycerin and when compared with SNP, mortality was lower in patients receiving clevidipine. Although Prowroznyk *et al*., reported an equal efficacy when comparing SNP with clevidipine for BP control following coronary artery surgery, hemodynamic changes including tachycardia and decreases in stroke volume were less with clevidipine.[9]

In our 3 patients, we found that clevidipine in doses ranging from 2-8 mg/hr effectively controlled perioperative BP without excessive hypotension or clinically significant rebound tachycardia. Given its short half-life, clevidipine can be rapidly titrated to achieve the desired BP during the perioperative period when changing levels of sympathetic stimulation related to pain, the surgical procedure, or concomitant use of anesthetic agents may occur. Should inadvertent hypotension occur, its short duration of action offers an additional advantage over nicardipine. The preliminary clinical data have demonstrated a limited effect on preload and cardiac output as well as only a mild increase in HR. Clevidipine is supplied in a concentration of 0.5 mg/mL in 50 or 100 mL vials. Because of solubility issues, it is provided in a lipid solution and is contraindicated in patients with allergy to eggs, egg products, soy beans or soy products as well as disorders of lipid metabolism. Although pricing varies according to region and supplier, the 100 mL vial is approximately $145. Given its efficacy in the adult cardiac population, future trials in the neurosurgical population appear warranted.
